# Female emancipation in a male dominant, sexually dimorphic primate under natural conditions

**DOI:** 10.1371/journal.pone.0249039

**Published:** 2021-04-19

**Authors:** Patrícia Izar, Marcelo Fernández-Bolaños, Lauren Seex, Gerrit Gort, Priscila Suscke, Marcos Tokuda, Olívia Mendonça-Furtado, Michele P. Verderane, Charlotte K. Hemelrijk

**Affiliations:** 1 Department of Experimental Psychology, University of São Paulo, São Paulo, Brazil; 2 Theoretical Research in Evolutionary Life Sciences, TRÊS, Groningen Institute for Evolutionary Life Sciences, University of Groningen, Groningen, Netherlands; 3 Biometris, Wageningen University & Research, Wageningen, Netherlands; 4 Parque Zoológico Municipal Quinzinho de Barros, Sorocaba, Brazil; 5 National Institute of the Atlantic Forest, Santa Teresa, Brazil; University of Iowa, UNITED STATES

## Abstract

In most group-living animals, a dominance hierarchy reduces the costs of competition for limited resources. Dominance ranks may reflect prior attributes, such as body size, related to fighting ability or reflect the history of self-reinforcing effects of winning and losing a conflict (the winner-loser effect), or both. As to prior attributes, in sexually dimorphic species, where males are larger than females, males are assumed to be dominant over females. As to the winner-loser effect, the computational model DomWorld has shown that despite the female’s lower initial fighting ability, females achieve some degree of dominance of females over males. In the model, this degree of female dominance increases with the proportion of males in a group. This increase was supposed to emerge from the higher fraction of fights of males among themselves. These correlations were confirmed in despotic macaques, vervet monkeys, and in humans. Here, we first investigate this hypothesis in DomWorld and next in long-term data of 9,300 observation hours on six wild groups of robust capuchin monkeys (*Sapajus libidinosus*; *S*. *nigritus*, and *S*. *xanthosternos*) in three Brazilian sites. We test whether both the proportion of males and degree of female dominance over males are indeed associated with a higher relative frequency of aggression among males and a higher relative frequency of aggression of females to males. We confirm these correlations in DomWorld. Next, we confirm in empirical data of capuchin monkeys that with the proportion of males in the group there is indeed an increase in female dominance over males, and in the relative frequency of both male-male aggression and aggression of females to males and that the female dominance index is significantly positively associated with male male aggression. Our results reveal that adult sex ratio influences the power relation between the sexes beyond predictions from socioecological models.

## Introduction

The concept of dominance was introduced by Schjelderup-Ebbe [1] to describe unidirectional aggressive behavior within pairs of animals in the social organization of domestic chickens. A dominance hierarchy can be deduced by combining all asymmetric relations of the pairs of individuals [2, 3]. In most group-living animals, a dominance hierarchy reduces the frequency of aggression by formalizing social relations, thus diminishing the costs of contest competition for limited resources [4, 5].

The proximate mechanisms involved in the formation of dominance hierarchies in animal groups has been subject to extensive investigation. In the traditional view a dominance hierarchy results from pre-existing, prior qualities, such as a larger body size [6–9] or higher levels of gonadotrophic hormones [10]. Rowell [11] proposed additionally that dominance ranks may reflect the history of the self-reinforcing effects of winning and losing competitive interactions [12–15]. Thus, an individual’s victory in a competitive interaction does not rely only on its superior pre-existing features. If an individual wins a dispute unexpectedly [e.g. even though it is smaller than its opponent), this victory may remain in the memory of both participants and influence subsequent encounters. Thus, in dominance hierarchies, both the initial intrinsic fighting abilities and the history of fighting outcomes have an effect on rank acquisition [16, 17]. In addition, asymmetry in dyadic relationships may also be caused by factors other than fighting abilities, such as the possession of a particular knowledge (e.g. in navigation, [18]), or a commodity that cannot be taken by force (such as a female’s fertilizable egg, [19]). Coalitionary support may also affect rank acquisition, such as the inheritance of maternal rank observed in cercopithecines and hyenas [20, 21].

In primates, the self-reinforcing effects of winning and losing fights have been shown in the past by Mendoza & Barchas [22] and recently, by several authors using Elo-rating. This method infers dominance while explicitly incorporating the effects of prior history on them (e.g. [23, 24]). Most studies have, however, investigated dominance relations only among individuals of the same sex (e.g. [25–27]) because, from the evolutionary point of view of socio-ecological hypotheses, individuals compete for resources mainly with others of the same sex. Males compete mainly for opportunities to mate and females mainly for food [28–30]. Like for mammals in general, in primates, the competition among males over access to reproductive partners is supposed to have resulted in the evolution of greater body mass and larger canine size in males than females [31]. In line with the view that dominance is related to prior attributes for fighting abilities, in these sexually dimorphic species, including humans, males are considered dominant over females [19, 32, 33]. Intersexual competition has however, rarely been investigated [34].

Yet, a few studies show intersexual dominance is evolutionary meaningful [19]; for example, females may compete with males for access to food, as in several species of capuchin monkeys (genus *Sapajus* [35, 36], genus *Cebus* [37]). Female dominance over males may be a strategy to resist sexual coercion by males [38, 39] and a guarantee for female mate choice [40]. Female dominance over males is found more typically in species where males and females have equal body size, such as lemurs [41] and marmosets (*Callithrix* spp. [42]). In species with male-biased sexual dimorphism in body size, female dominance may depend on specific circumstances. In bonobos (*Pan paniscus*), for instance, males may refrain from aggression to females in the reproductive context. This increases the dominance of females over them, and is considered evidence for the docile male hypothesis [43]. In vervet monkeys (*Chlorocebus pygerythrus*), dominance of females over males may relate to the females’ affiliative relationships with males [44] or depend on the self-reinforcing effects of winning and losing fights in combination with the adult sex ratio [45]. In these cases inter-sexual dominance does not exclusively depend on the greater prior fighting abilities of males than females despite their presence in these species, as is the case also for humans [19, 32, 33].

The idea that inter-sexual dominance in a species with male-biased sexual dimorphism may be affected by the self-reinforcing effects of winning and losing fights has been proposed by Hemelrijk and collaborators [46] using the computational model DomWorld. The self-reinforcing effects imply that after losing a fight, the chance to lose again increases, and after winning a fight, the chance to win again is enhanced [47]. Hemelrijk et al. [48] showed in this model that if the intensity of aggression is high (representing chasing and biting rather than just threats and slaps) the higher the proportion of males in a group, the higher the degree of dominance of females over males becomes. They explained this as follows. In groups with a greater proportion of males, males were in conflict relatively more often with other males (from all their conflicts with adults) than in groups with a lower proportion of males, and so they lost from other males more often. Therefore, these males sank low in rank, and females, in spite of their smaller body size, thus beat males relatively more often. Yet, the authors did not test in the model DomWorld these correlations between the proportion of males and both the proportion of male-male aggression and the proportion of female-male aggression as well as the correlation of female dominance with both factors (the proportion of male-male aggression and female male aggression). Therefore, in the present paper we first investigate whether these correlations hold in the model DomWorld.

The positive correlation between the degree of female dominance over males and the proportion of males in a group has been confirmed empirically in cercopithecoids, namely in macaques (*Macaca* spp. [48]) and recently, in vervet monkeys [45]. However, understanding the contribution of the winner-loser effect to the variation in primate intersexual dominance still requires a broader taxonomic knowledge [19].

Therefore, we test the hypothesis that female dominance over males increases with the proportion of males in a group in robust capuchin monkeys and also test its associated patterns in the form of the correlations mentioned above. The robust capuchin monkeys present an ideal case for testing the relation between sex ratio and female dominance [48], since a) the species-specific dominance of one sex over the other is incomplete [35, 36], b) groups show great intraspecific variation in adult sex ratio variation [49, 50], c) it is a phylogenetically independent clade, and d) we have long-term data on 6 groups of three wild populations of robust capuchin monkeys: *Sapajus nigritus*, *S*. *xanthosternos*, and *S*. *libidinosus*. Partial dominance of females over males in robust capuchin monkeys is remarkable, because their sexual dimorphism in body size is large [51]; in wild *S*. *libidinosus*, average female body mass is 2.1 kg and male is 3.5 kg [52].

We predict a positive correlation between the degree of female dominance and the proportion of males in the three species. In addition, we investigate both in the computational model DomWorld and in the empirical data whether the proportion of males was associated with a higher proportion of 1) male-male aggression and 2) female-male aggression, and whether female dominance was related to an increase in 1) proportion of male-male aggression and 2) female-male aggression. For comparability, we set the group size and sex ratio in the model to the values of the empirical data.

## Materials and methods

### The computational model DomWorld

We studied the processes underlying the correlation between the degree of female dominance and the proportion of males in a group in the computational model, DomWorld, for the same parameters as in earlier models [48], apart from taking the group size and composition like in the empirical data. In the model DomWorld, individuals group and compete while experiencing the self-reinforcing effects of winning and losing fights. Here, an individual groups (if it sees others at medium distance, in NearView, and maximum distance, MaxView, Table 1) and competes if others are too close to it (in Perspace). It starts a potential agonistic interaction with a mental battle, estimating whether it will win (number of mental battles, see Table 1). If it thinks it will win, it starts a real battle in which its chance to win depends on its relative dominance value to its opponent. After the outcome has been decided, the winner chases the loser (ChaseDist), the loser flees (FleeDist) and the dominance values are updated such that the value of the winner is increased and of the loser is decreased by the same amount. In the model, the dimorphism of the sexes is loosely reflected by the initial dominance value (Initial Dominance) and the intensity of aggression (StepDom), which are greater in males than females (see for parameters, Table 1, and for further information see [12, 48]).

**Table 1:** Parameters for DomWorld for females and males.

Parameter	Females	Males
Initial Dominance	16	32
StepDom	0.1	1
Number of mental battles	1	1
Field of View	120	120
PersSpace	4	4
NearView	24	24
MaxView	48	48
FleeDist	2	2
WithdrawDist	0	0
ChaseDist	1	1
MoveDist	1	1
WiggleTurn	0	0
WiggleTurnError	10	10
SearchTurn	90	90
SearchTurnError	10	10
WonTurn	0	0
WonTurnError	0	0
FleeTurn	180	180
FleeTurnError	10	10

Field of View indicates the viewangle with which individuals see others, MoveDist indicates step size, WiggleTurn and WiggleTurnError indicate small random turn during forward motion. Individuals turn after searching for others (SearchTurn with SearchTurnError), after winning a fight (WonTurn with WonTurnError) and after fleeing (FleeTurn with FleeTurnError), for the other parameters see main text and Hemelrijk [12, 48].

### Study populations

We used long-term data, namely 9,306 hours of observations, collected for three populations of capuchin monkeys in Brazil, *Sapajus libidinosus* in Fazenda Boa Vista, *S*. *nigritus* in Parque Estadual Carlos Botelho, and *S*. *xanthosternos* in Reserva Biológica de Una (Table 1). Fazenda Boa Vista is an area located in the Ecotone between Cerrado (Brazilian savannah) and Caatinga (semi-arid), in the state of Piauí, 9° 39’S 45° 25W (see [53] for a better description). Parque Estadual Carlos Botelho is an area of montane Atlantic Forest, located in southeastern Brazil, 24°00’ to 24°15’S 47°45’ to 48°10’W (see [54] for a better description). Reserva Biológica de Una is an area of lowland Atlantic Forest, in the northeastern Brazil, 15° 10’S 39° 03’W, and is a mosaic of mature forest with patches of plantations (see [55] for a better description). We studied two habituated groups in Fazenda Boa Vista (Chicão, hereafter CH, and Zangado, ZA), three groups in Parque Estadual Carlos Botelho (Laranja, LAR, Pimenta, PIM, and Testa, TES), and one group in Reserva Biológica de Una (Príncipe, PRIN) (Table 2). All adult group members included in this study were individually recognized by the observers.

**Table 2:** Study sites and study groups names, group composition, period of study and total hours of observation.

Study site	Study group	Study period	Number of adult group members Males: Females	Hours
FBV	CH	February 2007 to July 2007	2:4	215
FBV	CH	August 2007 to April 2008	2:5	327
FBV	CH	January 2009 to March 2010	4:4	691
FBV	CH	April 2010 to August 2010	4:4	402
FBV	ZA	April to May 2006	5:4	295
FBV	ZA	May 2006 to July 2006	1:4	100
FBV	ZA	August 2006 to April 2008	1:3	1171
FBV	ZA	January 2009 to December 2010	4:4	733
PECB	LAR	November 2001 to December 2002	2:4	805
PECB	PIM	January 2009 to August 2010	2:5	516
PECB	TES	January 2009 to August 2010	5:7	380
UNA	PRIN	December 2011 to July 2012	6:10	1050
UNA	PRIN	August 2012 to March 2013	6:10	1075
UNA	PRIN	April 2015 to March 2016	4:7	1308

FBV = Fazenda Boa Vista; PECB = Parque Estadual Carlos Botelho; UNA = Reserva Biológica de Una.

During the study period, the groups in Fazenda Boa Vista and Reserva Biológica de Una underwent changes in demography, experienced rank reversals, or both. Therefore, we cut the study period of group CH in five periods, of group ZA in four periods, and group PRIN in three periods, corresponding to different demographics. For the three groups in Parque Estadual Carlos Botelho, we had one period per study group (hereafter group-period). Before conducting the dominance analysis (see below), we examined the number of agonistic interactions per group-period. Here we only included group-periods if on average each individual had more than one interaction. We therefore excluded one period of group CH and analyzed 14 group-periods (Table 2).

### Observational methods and data collection

In all three areas, groups were followed from dawn to dusk, for 6–21 days per month, and monkeys behavior was recorded by two to three observers through 10-minute scan samples and records of all-occurrences of agonistic events among group members, identifying the context of the dispute and the individuals involved; new observers were trained by the previous ones until reaching 80% reliability (see [50] for details). We only included the records of agonistic interactions that were dyadic and of which the outcome was decided. Interactions were decided when the winner showed physical aggression (hitting, biting, or pushing), chased the opponent, or threatened it (e.g. open mouth threat display), and the receiver emitted signs such as submissive calls and/or facial and body displays (e.g. bared teeth display, cowering), and/or fleeing upon the arrival of the dominant one [35, 49]. In this study we tested a hypothesis about factors related to female dominance based on DomWorld, and DomWorld does not include explicit or intentional support. Therefore, agonistic events involving coalitions were not included in this study.

### Determination of dominance hierarchies in capuchin monkeys

Using the records of agonistic interactions, we generated a matrix of decided agonistic interactions for each group-period. From the matrices, we determined the dominance hierarchy of each group-period by ranking individuals on the basis of their average dominance index (ADI), which is their average fraction of winning from each opponent [56]. An identical hierarchy is obtained by the normalised David Score, but the method of calculation of the average proportion of winning is more easy and better understandable [56, 57], it is robust compared to others [56] and it does not ask for any arbitrary parameters, such as the k-value for representing the intensity of aggression [26], necessary on Elo-rating. The average dominance index (ADI) is calculated per individual as the average proportion with which the individual wins fights from all its interaction partners, thus, whenever it did not interact at all with a certain partner, this partner was excluded from the calculation of its average. In our sample of 14 group-periods, the proportion of dyads with unknown dominance relations due to the absence of interactions varied from zero to 39% (Table 3). The proportion of unknown relations was moderately correlated with group size (Pearson correlation, r = 0.59, n = 14 p = 0.025, two-tailed), but not with the degree of female dominance or the proportion of males in the group. This calculation of the average dominance index was performed with the program Matrix Tester v223b developed by Hemelrijk and co-workers (available on request). The degree of female dominance was measured by the female dominance index (FDI) as the relative position of females versus males in the dominance hierarchy. It is calculated as the average for females of the proportion of males they are dominant over [45]. The female dominance index ranges between 0 (no female is dominant over a male) and 1 (all females are dominant over all males).

**Table 3:** Demography and dominance relations in empirical groups of robust capuchin monkeys.

Study site	Group	Period	proportion of males	Number of adults	Number of males	FD	MM ago	FM ago	Number of aggressive interactions	% unknown relations
FBV	CH	2007	0.33	6	2	0.13	0.33	0.33	21	23.30
FBV	CH	2007/08	0.29	7	2	0.40	0.15	0.09	60	21.40
FBV	CH	2009/2010	0.50	8	4	0.63	0.44	0.76	85	3.60
FBV	CH	2010 b	0.50	8	4	0.72	0.68	0.57	60	21.40
FBV	ZA	2006 a	0.56	9	5	0.73	0.88	0.48	67	18.00
FBV	ZA	2006 b	0.20	5	1	0.00	0.00	0.00	10	25.00
FBV	ZA	2006/2008	0.25	4	1	0.00	0.00	0.00	28	0.00
FBV	ZA	2009/2010	0.50	8	4	0.50	0.31	0.93	55	13.90
PECB	LAR	2002	0.33	6	2	0.44	0.78	0.13	34	16.70
PECB	PIM	2010	0.29	7	2	0.15	0.75	0.00	28	28.60
PECB	TES	2010	0.42	12	5	0.53	0.81	0.20	26	39.40
UNA	PRIN	2012	0.38	16	6	0.13	0.39	0.64	58	33.30
UNA	PRIN	2013	0.38	16	6	0.23	0.46	0.63	55	36.70
UNA	PRIN	2016	0.36	11	4	0.33	0.37	0.44	41	31.80

Proportion of males, number of adults and number of males are shown per group. FD = female dominance over males; period is the year of study; MM ago = male-male aggression per total male aggression; FM ago = female-male aggression per total female aggression; % unknown relations = the number of dyads with zero dominance interactions over all possible dyads in a group. FBV = Fazenda Boa Vista; PECB = Parque Estadual Carlos Botelho; UNA = Reserva Biológica de Una.

We determined the dominance hierarchies per each group-period (Table 3). Rates reported here reflect dyadic decided agonistic events only among adults normalized by total observation hours for each period of each group.

Field data collection was conducted under the following Brazilian government permits: SMA #002.534/2008 (*S*. *nigritus*, PECB); SisBio # 47501 (*S*. *xanthosternos*, ReBio UNA), and SisBio # 28689 (*S*. *libidinosus*; Fazenda Boa Vista, Gilbués). Data collection for the research followed was reviewed and approved by The Ethics Committee of the Psychology Institute of University of São Paulo—CEUA # 6870180216, in accord with the Brazilian legislation (law #11.794, October 8, 2008).

### Statistical analyses

We investigated the relation between the degree of female dominance (FDI) and the proportion of males in a group in the empirical data using a generalized linear mixed model (GLMM). We assumed the betabinomial distribution for the female dominance index, relating the expected dominance index to the proportion males, using a logit link function, and introducing random population effects and group-within-population effects to handle repeated observations on the same group (over different periods). The total n for the betabinomial distribution is the product of the numbers of males and of females in a group, whereas the number of “successes” is the sum of the numbers of males dominated by the females. The betabinomial distribution allows for overdispersion compared to the ordinary binomial distribution. The model was fitted using the glmmTMB package [58] of R, version3.6.1 [59] and visualized in a plot of FDI versus proportion males, showing the fitted line with a 95% confidence band. Model diagnostics were obtained using the DHARMa package [60]. Pseudo R^2^ ‘s based on likelihoods were calculated using function r.squaredLR from package MuMIn [61]. We also ran a model to check whether the absolute number of males would explain the degree of female dominance better than the proportion of males per group.

To get an understanding of the associated processes, we ran GLMM’s again assuming the betabinomial distribution for the female dominance index to analyze its relation with male-male aggression in relation to total male aggression, and female-male aggression in relation to total female aggression both in the empirical data and data from the model DomWorld. In the GLMM for the empirical data random effects for populations and groups were included as before. We used data from 14 group-periods with the exception of analyses with regard to male-male aggression in relation to total male aggression, in which the two observations from the single-male group-periods ZA in Fazenda Boa Vista were excluded. For the tests in the DomWorld model we matched the group size and proportion of males to our empirical data and otherwise used our standard parameters from previous studies [48]. This resulted in 40 runs of 14 groups (S1 Table), fitting betabinomial GLMM’s to each of the 40 runs and averaging the coefficients.

Nested models were compared using likelihood ratio tests (LRT). Non-nested models were compared based upon AIC values. Regression coefficients with standard errors and P-values based upon Wald-tests were reported.

Additional analyses were made to investigate the relation between the variables included in the models, male-male aggression per total male aggression and female-male aggression per total female aggression, as responses, and the proportion of males in the group as regressor using GLMM’s with betabinomial distributions. These additional analyses were made considering the total sample of 14 groups for the female-male aggression per total female aggression and with the subset of 12 groups for the male-male aggression per total male aggression, excluding the two single-male groups.

## Results

### Computational model, DomWorld

The degree of dominance of females over males (FDI) was significantly positively related with the proportion of males in the group (40 runs of each of 14 groups of the same sex ratio as empirical data, mean slope β = 1.31 with s.e.m. = 0.47, t = 2.80, P = 0.008) when we fitted betabinomial GLMM’s with the proportion of males as a single regressor.

We confirmed statistically the suggested associated patterns [47], namely with a greater proportion of males in the group there was an increase of both, male-male aggression per total aggression by males (GLMM, 12 groups, mean slope β = 5.70 with s.e.m. = 0.21, t = 26.77, P<0.0001) and female-male aggression per total aggression by females (GLMM, 14 groups, mean slope β = 5.02 with s.e.m. = 0.19, t = 26.82, P<0.0001). Related to this, we confirmed that the female dominance over males (FDI) increased also with both male-male aggression per total aggression by males (GLMM, 12 groups, mean slope β = 3.68 with s.e.m. = 0.37, t = 9.98, P<0.0001) and female-male aggression per total aggression by females (GLMM, 14 groups, mean slope β = 3.97 with s.e.m. = 0.19, t = 20.63, P<0.0001).

### Empirical data

**Fig 1:**
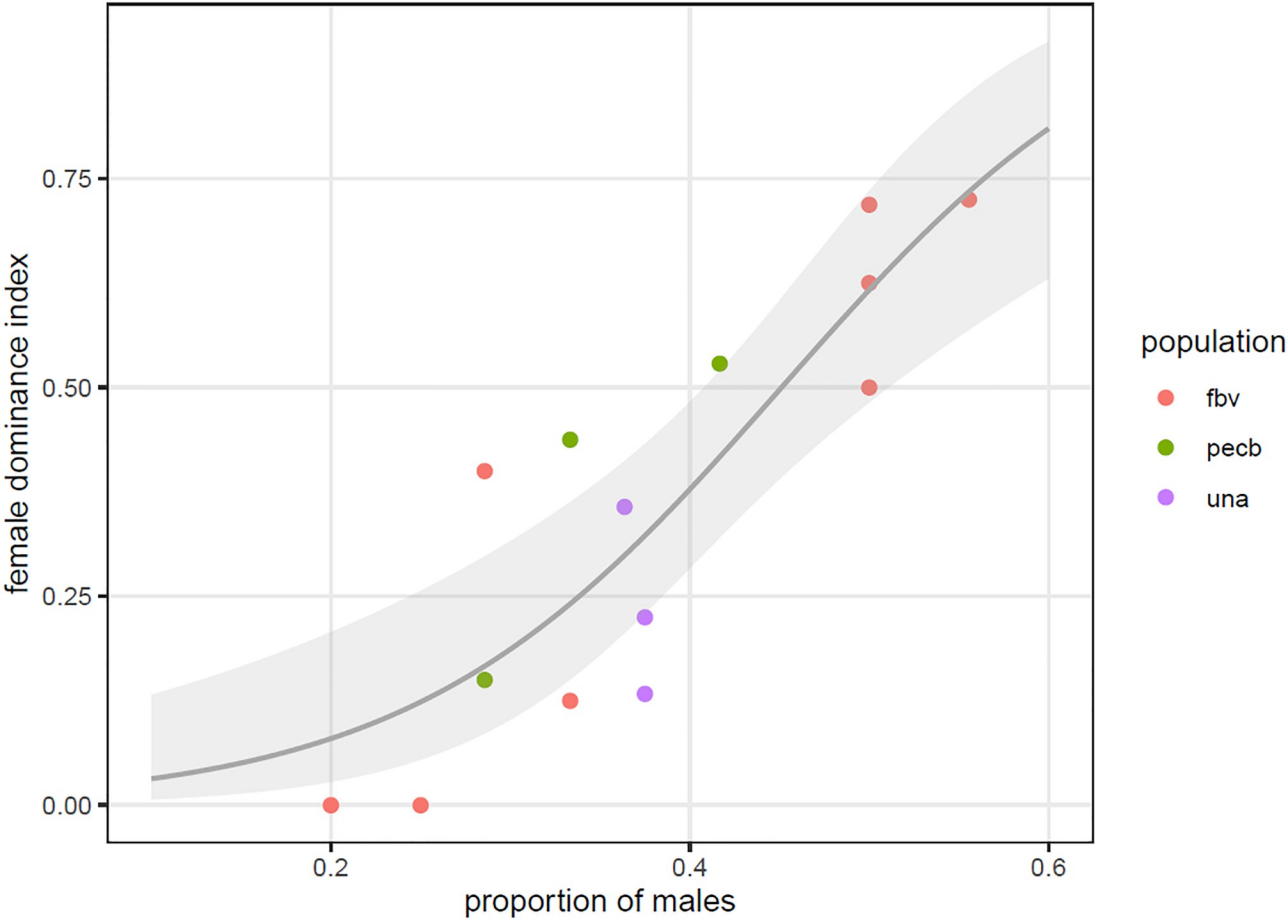
Degree of female dominance as a function of proportion of males per group in 14 hierarchies of robust capuchin monkeys from three wild populations. Degree of female dominance was measured as the proportion of males ranking below females on average (the female dominance index, FDI).

We tested the predictions of DomWorld in the empirical data. We confirmed a significant, positive association between the degree of female dominance over males (FDI) and the proportion of males in the group (GLMM, 14 group-periods, LRT X^2^ = 14.75 on 1 df, P = 0.00012; regression coefficient β = 9.74 with se = 2.34, z = 4.16, P<0.0001; Fig 1). The fitted model (compared to the intercept-only model) had a pseudo R^2^ equal to 0.66. This fraction was solely contributable to the sex-ratio, as the pseudo R^2^ for the fitted model compared to the model with random effects was 0.66 too. Residual plots did not reveal deviations from the assumed betabinomial model (see S1 Appendix). The betabinomial dispersion parameter was not significantly different from zero (LRT X^2^ = 1.51 on mixture of X^2^ distributions with 0 and 1 df, P = 0.11), and neither were the variance components for population and group (LRT X^2^ = 0.031 on mixture of X^2^ distributions with 0, 1 and 2 df, P = 0.68). The model with the absolute number of males in the group as regressor had a higher AIC value (AIC = 78.8) than the model with the proportion of males (AIC = 66.9), indicating a worse fit for the model with absolute number of males.

As to the associated patterns that were present in DomWorld, we confirmed that male-male aggression per total aggression by males was significantly positively related with the proportion of males in the group when considering 12 group-periods (hierarchies) excluding the two one-male groups (β = 7.26 with se = 2.78, z = 2.61, P = 0.009) and that female-male aggression per total aggression by females was significantly positively associated with the proportion of males in the group (14 group-periods, β = 11.35 with se = 3.04, z = 3.73, P = 0.0002). Testing the association between the female dominance index FDI and fraction male-male aggression of total male aggression, we found a significantly positive regression coefficient (N = 12 group-periods, slope β = 2.27 with se = 0.98, z = 2.31, P = 0.021). The association between FDI and female-male aggression per total aggression by females was positive but not significant (N = 14 group-periods, slope β = 1.50 with se = 1.00, z = 1.50, P = 0.13).

**Fig 2:**
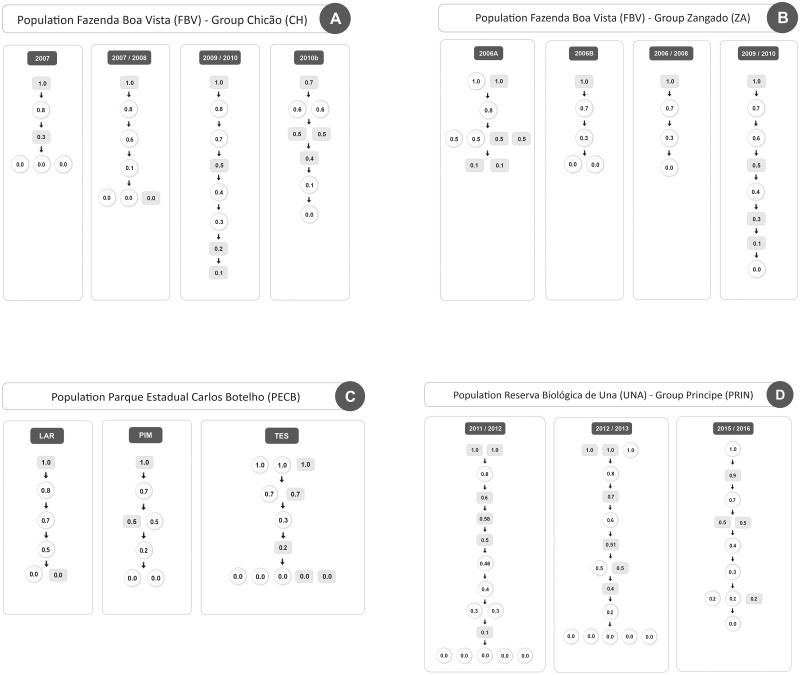
Schematic representation of dominance hierarchies based on the average dominance index (ADI) of members of both sexes of six groups of robust capuchin monkeys from three wild populations (values inside boxes are ADI; males in grey rectangles; females in white ellipses). A) Population of *Sapajus libidinosus* from Fazenda Boa Vista, group Chicão in 4 periods, B) Population of *Sapajus libidinosus* from Fazenda Boa Vista, group Zangado in 4 periods, C) Population of *Sapajus nigritus* from Parque Estadual Carlos Botelho, groups LAR, PIM and TES, one period, D) Population of *Sapajus xanthosternos* from Reserva Biológica de Una, group Príncipe, three periods. Study periods as shown in Table 3.

When looking at the individual hierarchies we see that in 13 of the 14 hierarchies a male was the most dominant individual of the group, even while in four of these hierarchies females were equally dominant to the alpha male in the group (Fig 2). Note that in the three populations there was at least one group-period where one or two females shared the alpha position with one or two males (Fazenda Boa Vista the group ZA in the period 2006a, Fig 2B, in Parque Estadual Carlos Botelho the group TES, Fig 2C, and Reserva Biológica de Una the group PRIN in the years 2013 and 2016, Fig 2D). Interestingly, groups with tied relations in the alpha position were not necessarily those with the higher female dominance indices, but those with an adult sex ratio equal or above 30% in all the populations of our study. In general in 5 hierarchies females were equally, or more, dominant than males in the group (female dominance index > = 0.5) (four in Fazenda Boa Vista, and one in Parque Estadual Carlos Botelho) (Table 3).

## Discussion

We confirmed in DomWorld the correlations related to the processes supposedly associated with the increase in dominance of females over males with the proportion of males in the group: a greater proportion of males in the group and greater dominance of females over males (FDI) were each significantly positively associated with a relatively (to their total number of fights with adults) higher frequency with which males fight each other and with which females attack males.

In our empirical data we show for the first time that in groups of a wild Neotropical primate the larger the proportion of males was in a group, the larger was the proportion of males that females dominated on average, in line with previous findings in cercopithecoids, namely macaques [48] and vervet monkeys [45]. As to the associated patterns in the empirical data, we confirmed that the proportion of males in the group was significantly positively associated with the relative aggression by males to males and with the relative aggression of females to males. This indicates that more aggression among males may have led to a stronger differentiation of the hierarchy among males and may have encouraged females to attack (low ranking) males more often as apparent from the higher relative frequency of fights by females to males, thus revealing their higher dominance relative to males. We also confirmed that the female dominance index was positively associated with the relative aggression of males to males, which suggests that more attacks among males results ia a stronger hierarchical differentiation to more dominance of females over males. The correlation between the female dominance index and the relative aggression of females to males was positively associated but non-significant. We attribute this to the small data-set. We thus confirm both in DomWorld and from empirical data from a Neotropical primate species that an increase in male-male aggression underlies the correlation between proportion of males and female dominance over males (FDI) and this female dominance may lead to more aggression of females to males.

We exclude that the observed correlation between the degree of female dominance over males and the proportion of males in wild groups of robust capuchin monkeys results from processes related to the docile male hypothesis, that is, females become more dominant over males because males are less aggressive to them [43]. Neither in the model DomWorld nor in our empirical data do we see that males decrease their frequency of aggression to females more the higher the fraction of males in the group beyond what we expect from random encounters (GLMM of frequency of male-female aggression, corrected for total frequency and fraction female opponents and explained by proportion males, showed in DomWorld data averaged over 40 repeats a positive relationship, average β = 0.22, sem = 0.058, t = 3.83, P = 0.00045; for empirical data β = -1.37, se = 1.42, z = -0.96, P = 0.34; for details see S1 Appendix).

One of the shortcomings of our analysis is that we did not analyze whether a higher degree of female dominance over males was associated with more coalitionary support received from males by females. Therefore, we cannot exclude the hypothesis that the degree of female dominance increases with the proportion of males in a group due to the females receipt of support from males when females are in conflict with other males. However, in the present study, we only included dyadic conflicts that did not involve coalitions, so we excluded any direct effect of support females received from males in agonistic events on female dominance rank. Whether these coalitionary events still contribute to female victories in dyadic encounters, should be investigated in future studies.

Showing in capuchin monkeys that females are sometimes dominant over males contributes to the discussion about the plasticity of dominance hierarchies and the proximate causes of intersexual dominance relations. In behavioral ecology, it is usually thought that the patterning of dominance relations is species-typical [62], obviously reflecting evolved cognitive features that currently affect how the individuals develop dominance relations [4]. Species are often classified as despotic or egalitarian, and dominance rank is commonly inferred from assumed correlates of fighting ability, such as body size [31]. In capuchin monkeys, while some studies have described intersexual dominance relations [3, 35–37], the most dominant individual is usually a male ([35, 49, 50, 63, 64], this study) and males have been generally considered dominant over females [19, 49]. Here we show that this pattern is flexible and contingent to the adult sex ratio. In some of our groups, or during certain periods, more than one individual of both sexes shared the alpha position in the hierarchy. Tied dominance relations have previously been described in the genus *Sapajus*, but only in lower ranks [3, 53].

In primates, the adult sex ratio has been shown to affect several aspects of primate behavior [33], including patterns of grouping (stable, fission-fusion, sometimes fission-fusion; [65], mating systems [66], and male-male affiliative patterns [67]). Consistent evidence that the variation in adult sex ratio is related to variation in patterns of intersexual agonistic relations was still lacking (review in [34]). Here, we have added evidence that demographic factors alter power relations between the sexes.

Our results offer a phylogenetically independent point to existing evidence that a higher proportion of males in the group results in more dominance of females over males, as shown in macaques and [48] in vervet monkeys [45]. This phylogenetic independence is relevant since phylogenetic conservatism may explain behavioral similarities in related taxa [68–70], as has been shown for several features of the social systems of the Cercopithecoidea [71]. We show these effects of self-organisation are independently of phylogeny. We also expand the limits of the phylogenetic distribution of female dominance over males and confirm that it may arise from an ancestor with a male-dominant structure [19].

## Supporting information

S1 Table Data generated by the computer model DomWorld. *The* first worksheet shows the parameters introduced in this model run. The next worksheets show the matrix of agonistic interactions between pairs of virtual individuals in each run in the model. Worksheets are named after the group composition. Figures represent the number of individuals, f = females and m = males.
(XLSX)

S1 Appendix Complete results of statistical analysis using Rmd script. (PDF)
