# Supplementary material for: Female emancipation in a male dominant, sexually dimorphic primate under natural conditions
Source: PLoS One. 2021 Apr 19;16(4):e0249039. doi: 10.1371/journal.pone.0249039 (PMC8055024; doi:10.1371/journal.pone.0249039)
Supplement: S1 Appendix — (PDF) [file pone.0249039.s002.pdf]

Female emancipation in a male dominant, sexually dimorphic primate under natural conditions

Patrícia Izar[*]
Marcelo Fernández-Bolaños[†]
Lauren Seex[‡]
Gerrit Gort[§]
Priscila Suscke[¶]
Marcos Tokuda[‖]
Olívia Mendonça-Furtado[**]
Michele P. Verderane[††]
Charlotte K. Hemelrijk[‡‡]

Load some packages

```
library(glmmTMB)
library(DHARMa)
library(MuMIn)
library(car)
library(ggplot2)
```

Read data (empirical data and DomWorld)

```
# Empirical data
C <- read.csv("Capuchins_final.csv", header=TRUE)
C$nadults <- C$nmales+C$nfemales
C$sexratio <- C$nmales / C$nadults
C
```

```
##     pop group  year   yr nmales nfemales groupsize       FDI FDI.num
## 1   fbv    CH  2007 2007      2        4        18 0.1250000     1.0
## 2   fbv    CH  2008 2008      2        5        19 0.4000000     4.0
## 3   fbv    CH  2010 2010      4        4        21 0.6250000    10.0
## 4   fbv    CH 2010b 2010      4        4        21 0.7187500    11.5
## 5   fbv    ZA 2006a 2006      5        4        14 0.7250000    14.5
## 6   fbv    ZA 2006b 2006      1        4        10 0.0000000     0.0
## 7   fbv    ZA  2007 2007      1        3        10 0.0000000     0.0
## 8   fbv    ZA  2010 2010      4        4        17 0.5000000     8.0
## 9  pecb   LAR  2002 2002      2        4        13 0.4375000     3.5
## 10 pecb   PIM  2010 2010      2        5        15 0.1500000     1.5
## 11 pecb   TES  2010 2010      5        7        21 0.5285714    18.5
## 12  una  PRIN  2012 2012      6       10        33 0.1333333     8.0
## 13  una  PRIN  2013 2013      6       10        37 0.2250000    13.5
## 14  una  PRIN  2016 2016      4        7        30 0.3571429    10.0
##    FDI.denom MMago MFago FMago FFago nadults  sexratio
```

[*]Department of Experimental Psychology, University of Sao Paulo, Brazil
[†]Department of Experimental Psychology, University of Sao Paulo, Brazil
[‡]Theoretical Research in Evolutionary Life Sciences, TRÊS, Groningen Institute for Evolutionary Life Sciences, University of Groningen, Netherlands
[§]Biometris, Wageningen University and Research, Wageningen, The Netherlands
[¶]Department of Experimental Psychology, University of São Paulo, Brazil
[‖]Parque Zoológico Municipal Quinzinho de Barros, Sorocaba, Brazil
[**]Department of Experimental Psychology, University of São Paulo, Brazil
[††]Department of Experimental Psychology, University of Sao Paulo, Brazil
[‡‡]Theoretical Research in Evolutionary Life Sciences, TRÊS, Groningen Institute for Evolutionary Life Sciences, University of Groningen, Netherlands

```
## 1          8     4     8     3     6       6 0.3333333
## 2         10     4    23     3    30       7 0.2857143
## 3         16    17    22    35    11       8 0.5000000
## 4         16    25    12    13    10       8 0.5000000
## 5         20    35     5    13    14       9 0.5555556
## 6          4     0     4     0     6       5 0.2000000
## 7          3     0     9     0    19       4 0.2500000
## 8         16     8    18    27     2       8 0.5000000
## 9          8     6     3    13    12       6 0.3333333
## 10        10    12     4     0    12       7 0.2857143
## 11        35    17     4     1     4      12 0.4166667
## 12        60    17    27     9     5      16 0.3750000
## 13        60    21    25     5     4      16 0.3750000
## 14        28    10    15     7     9      11 0.3636364
```

The empirical dataset consists of data on 14 group-year combinations, stemming from three populations (fbv, pecb, una) and groups followed over time within each population: within population fbv there are two groups (followed over time), within population pecb there are three groups (in single years) and within population una there is one group (in three years).

```
# Data from DomWorld
CDW <- read.csv("FemaleDominance_SummaryResults_allcalcFixed_June27_inc1malegroups_v3.csv")
# Total number of Capuchins in group:
# Denominator of dominance score is number of females times number of males
# (all females dominant to all males):
CDW$nfemales <- CDW$nadults - CDW$nmales
CDW$FDI.denom <- CDW$nfemales * CDW$nmales
CDW$FDI.num <- round(CDW$FDI.denom * CDW$FDI)
CDW$run <- as.factor(CDW$run)
head(CDW)
```

```
##   run sexratio nadults nmales       FDI mmagopermale fmagopermale
## 1   1     0.25       4      1 0.6666667           NA          289
## 2   2     0.25       4      1 1.0000000           NA          441
## 3   3     0.25       4      1 0.0000000           NA          195
## 4   4     0.25       4      1 0.6666667           NA          301
## 5   5     0.25       4      1 0.0000000           NA           78
## 6   6     0.25       4      1 1.0000000           NA          317
##   mfagopermale ffagoperfem mfagoperfem fmagopertotfemaleago
## 1          226    191.6667    75.33333            0.3344907
## 2          113    174.6667    37.66667            0.4569948
## 3          339    180.6667   113.00000            0.2645862
## 4          235    189.0000    78.33333            0.3467742
## 5          538    152.0000   179.33333            0.1460674
## 6          226    193.3333    75.33333            0.3534002
##   mmagopertotmaleago       covf covm       prop1 prop2 fmago ffago mmago
## 1                 NA 0.24838504   NA 0.338817261    NA    63   575     0
## 2                 NA 0.03677845   NA 0.457482064    NA   161   524     0
## 3                 NA 0.41436750   NA 0.263444848    NA    31   542     0
## 4                 NA 0.31626068   NA 0.355902857    NA    64   567     0
## 5                 NA 0.35775606   NA 0.143982725    NA    33   456     0
## 6                 NA 0.16422817   NA 0.360528634    NA   100   580     0
##   mfago group nfemales FDI.denom FDI.num
## 1   226     1        3         3       2
## 2   113     1        3         3       3
## 3   339     1        3         3       0
## 4   235     1        3         3       2
## 5   538     1        3         3       0
## 6   226     1        3         3       3
```

DomWorld data consist of 40 simulated runs, each mimicking the empirical data with respect to the number of groups (n=14) and proportions of males.

Female Dominance Index and the (beta)binomial distribution

The Female Dominance Index (FDI) may be looked upon as a (possibly overdispersed) binomial fraction. To see this, consider the following. For female i in a group the number of submissive males y_i out of n_M males is counted. For y_i it seems reasonable to assume that $y_i \sim Bin(n_M, p_i)$ with p_i the probability that a male is submissive to female i. For all females in the group together the total number of dominated males is $y = \sum y_i$. For y a binomial distribution with binomial total $n_F * n_M$ and probability $\bar{p}_.$ comes to mind, with $\bar{p}_.$ the average of the individual female's probabilities to dominate a male. Because the individual probabilities are not constant and counts are not independent, a simple binomial distribution is likely not valid. Therefore, we consider the more general betabinomial distribution, which allows for overdispersion compared to the binomial distribution. The FDI is just the (beta)binomial fraction as just described: $y/(n_F * n_M)$.

Analysis of DomWorld data: GLMM's

DomWorld data, 40 runs, with group sizes and male proportions as in empirical data.

For each of the 40 runs we fit the following GLMM's:

1) betabinomial response FDI, regressor sexratio
2) betabinomial response fraction mmago per total male ago, regressor sexratio
3) betabinomial response fraction fmago per total female ago, regressor sexratio
4) betabinomial response FDI, regressor mmagopertotmaleago
5) betabinomial response FDI, regressor fmagopertotfemaleago
6) negative binomial response mfago, offset total male ago and fraction female opponents, regressor sexratio.

In models 1-5 we use a betabinomial distribution for the response, together with a logit-link function. In models 1, 4 and 5 the response is the FDI, using a numerator and denominator as discussed in the previous section. In model 2 the betabinomial response is the proportion male-male aggression, i.e. the frequency of male-male aggression compared to the frequency of all male aggression towards adults (regardless of the gender). In model 3 it is the proportion female-male aggression, i.e. the frequency of female-male aggression compared to the frequency of all female aggression towards adults.

In model 6 we check whether there are signs supporting the Docile Male Hypothesis. For this, we relate the frequency of male-female aggression to the proportion males, but now correcting for the total frequency of male aggression and the proportion of female opponents of the aggressor. We do this using a GLMM for the frequency of male-female aggression, assuming a negative binomial distribution and a log link function. In the fixed part of this model we not only allow for an effect of proportionof males, but we also add an offset depending on the frequency of all male aggression and the proportion of female opponents.

To clarify this approach, take the following example. Assume we have a group with 4 males and 4 females, where the 4 males direct 10 agonistic activities towards females, on a total of 40 agonistic activities. For each acting male there are 4 females and 3 other males, so that, if gender would not play a role, 40 * (4/7) = 22.9 agonistic activities are expected to be directed towards females. In this example there are only 10, which is less than expected "by chance". With the GLMM we check whether the frequency of male-female aggression, corrected for total male aggression and female proportion through an offset, depends on the proportion of males in the group. The Docile Male Hypothesis suggests that in groups with larger proportions of males, the corrected male-female aggression decreases.

In the analysis of the DomWorld data GLMM's are fitted per run. Next, the relevant regression coefficients are averaged over the 40 runs, and t-tests are performed.

```
resultsDW.fit <- matrix(nrow=40, ncol=24)
colnames(resultsDW.fit) <-
  c("b.FDI.sr","se.FDI.sr","z.FDI.sr","P.FDI.sr",
    "b.mm.sr","se.mm.sr","z.mm.sr","P.mm.sr",
    "b.fm.sr","se.fm.sr","z.fm.sr","P.fm.sr",
    "b.FDI.mm","se.FDI.mm","z.FDI.mm","P.FDI.mm",
    "b.FDI.fm","se.FDI.fm","z.FDI.fm","P.FDI.fm",
    "b.mfc.sr","se.mfc.sr","z.mfc.sr","P.mfc.sr")

resultsDW.fit <- as.data.frame(resultsDW.fit)

CDW$offsmm <- log((CDW$mmago+CDW$mfago)*((CDW$nmales-1)/(CDW$nadults-1)))
CDW$offsmf <- log((CDW$mmago+CDW$mfago)*(CDW$nfemales/(CDW$nadults-1)))

for (r in 1:40) {
  CDWr <- CDW[CDW$run==r,]

  CDWr.mm <- CDWr[CDWr$nmales>1,]

  glmmTMB.DWfs <- glmmTMB(cbind(FDI.num, FDI.denom) ~ sexratio,
                          family=betabinomial, data=CDWr)  #n=14
  resultsDW.fit[r, 1:4] <- coef(summary(glmmTMB.DWfs))$cond[2,]

  glmmTMB.DWmms <- glmmTMB(cbind(mmago, mfago) ~ sexratio,
```

```
                      family=betabinomial, data=CDWr.mm)   #n=12
  resultsDW.fit[r, 5:8] <- coef(summary(glmmTMB.DWmms))$cond[2,]

  glmmTMB.DWfms <- glmmTMB(cbind(fmago, ffago) ~ sexratio,
                      family=betabinomial, data=CDWr)  #n=14
  resultsDW.fit[r, 9:12] <- coef(summary(glmmTMB.DWfms))$cond[2,]

  glmmTMB.DWfmm <- glmmTMB(cbind(FDI.num, FDI.denom) ~ mmagopertotmaleago,
                      family=betabinomial, data=CDWr.mm) #n=12
  resultsDW.fit[r,13:16] <- coef(summary(glmmTMB.DWfmm))$cond[2,]

  glmmTMB.DWffm <- glmmTMB(cbind(FDI.num, FDI.denom) ~ fmagopertotfemaleago,
                      family=betabinomial, data=CDWr)  #n=14
  resultsDW.fit[r,17:20] <- coef(summary(glmmTMB.DWffm))$cond[2,]

  glmmTMB.DWmfcs <- glmmTMB(mfago ~ offset(offsmf) + sexratio,
                      family=nbinom2(link="log"), data=CDWr.mm)  #n=12
  resultsDW.fit[r,21:24] <- coef(summary(glmmTMB.DWmfcs))$cond[2,]

}

par(mfrow=c(3,2))

#FDI explained by sexratio
mean(resultsDW.fit$b.FDI.sr)
```

```
## [1] 1.309906
```

```
t.test(resultsDW.fit$b.FDI.sr)
```

```
##
##  One Sample t-test
##
## data:  resultsDW.fit$b.FDI.sr
## t = 2.7995, df = 39, p-value = 0.007918
## alternative hypothesis: true mean is not equal to 0
## 95 percent confidence interval:
##  0.3634709 2.2563411
## sample estimates:
## mean of x
##  1.309906
```

```
(se.b <- sd(resultsDW.fit$b.FDI.sr)/sqrt(length(resultsDW.fit$b.FDI.sr)))
```

```
## [1] 0.4679089
```

```
hist(resultsDW.fit$b.FDI.sr, breaks=seq(-6,10,2), xlab="slope sexratio",
     main="FDI: slope sexratio (DW)")
(emp.res <- coef(summary(glmmTMBo1))$cond[2,1])
```

```
## [1] 9.736143
```

```
abline(v=emp.res, col="red",lwd=2)
text(x=emp.res-2, y=12, labels="empirical result", col="red")

#fraction male-male aggression explained by sex ratio
mean(resultsDW.fit$b.mm.sr)
```

```
## [1] 5.695858
```

```
t.test(resultsDW.fit$b.mm.sr)
```

```
##
##  One Sample t-test
```

```
##
## data:  resultsDW.fit$b.mm.sr
## t = 26.768, df = 39, p-value < 2.2e-16
## alternative hypothesis: true mean is not equal to 0
## 95 percent confidence interval:
##  5.265456 6.126261
## sample estimates:
## mean of x
##  5.695858
```

```
(se.b <- sd(resultsDW.fit$b.mm.sr)/sqrt(length(resultsDW.fit$b.mm.sr)))
```

```
## [1] 0.2127872
```

```
hist(resultsDW.fit$b.mm.sr, xlab="slope sex ratio",
     main="frac MM aggr: slope sex ratio (DW)")
(emp.res <- coef(summary(glmmTMBe1))$cond[2,1])
```

```
## [1] 7.262394
```

```
abline(v=emp.res, col="red",lwd=2)
text(x=emp.res, y=12, labels="empirical result", col="red")


#fraction female-male aggression explained by sex ratio
mean(resultsDW.fit$b.fm.sr)
```

```
## [1] 5.021726
```

```
t.test(resultsDW.fit$b.fm.sr)
```

```
##
##  One Sample t-test
##
## data:  resultsDW.fit$b.fm.sr
## t = 26.82, df = 39, p-value < 2.2e-16
## alternative hypothesis: true mean is not equal to 0
## 95 percent confidence interval:
##  4.643002 5.400451
## sample estimates:
## mean of x
##  5.021726
```

```
(se.b <- sd(resultsDW.fit$b.fm.sr)/sqrt(length(resultsDW.fit$b.fm.sr)))
```

```
## [1] 0.187238
```

```
hist(resultsDW.fit$b.fm.sr, xlim=c(2,12), xlab="slope sex ratio",
     main="frac FM aggr: slope sex ratio (DW)")
(emp.res <- coef(summary(glmmTMBe2))$cond[2,1])
```

```
## [1] 11.34573
```

```
abline(v=emp.res, col="red",lwd=2)
text(x=emp.res-1, y=15, labels="empirical result", col="red")


#FDI explained by male-male aggression
mean(resultsDW.fit$b.FDI.mm)
```

```
## [1] 3.678568
```

```
t.test(resultsDW.fit$b.FDI.mm)
```

```
##
##  One Sample t-test
```

```
##
## data:  resultsDW.fit$b.FDI.mm
## t = 9.9763, df = 39, p-value = 2.737e-12
## alternative hypothesis: true mean is not equal to 0
## 95 percent confidence interval:
##  2.932739 4.424397
## sample estimates:
## mean of x
##  3.678568
```

```
(se.b <- sd(resultsDW.fit$b.FDI.mm)/sqrt(length(resultsDW.fit$b.FDI.mm)))
```

```
## [1] 0.368731
```

```
hist(resultsDW.fit$b.FDI.mm, xlab="slope fraction male-male aggression",
     main="FDI: slope fraction MM aggr (DW)")
emp.res <- coef(summary(glmmTMBa1))$cond[2,1]
abline(v=emp.res, col="red",lwd=2)
text(x=emp.res, y=19, labels="empirical result", col="red")


#FDI explained by female-male aggression
mean(resultsDW.fit$b.FDI.fm)
```

```
## [1] 3.972739
```

```
t.test(resultsDW.fit$b.FDI.fm)
```

```
##
##  One Sample t-test
##
## data:  resultsDW.fit$b.FDI.fm
## t = 20.627, df = 39, p-value < 2.2e-16
## alternative hypothesis: true mean is not equal to 0
## 95 percent confidence interval:
##  3.583172 4.362306
## sample estimates:
## mean of x
##  3.972739
```

```
(se.b <- sd(resultsDW.fit$b.FDI.fm)/sqrt(length(resultsDW.fit$b.FDI.fm)))
```

```
## [1] 0.1925984
```

```
hist(resultsDW.fit$b.FDI.fm, xlim=c(0,10), xlab="slope fraction female-male aggression",
     main="FDI: slope fraction FM aggr (DW)")
(emp.res <- coef(summary(glmmTMBa2))$cond[2,1])
```

```
## [1] 1.501159
```

```
abline(v=emp.res, col="red",lwd=2)
text(x=emp.res, y=14, labels="empirical result", col="red")


#corrected male-female aggression by sexratio
mean(resultsDW.fit$b.mfc.sr)
```

```
## [1] 0.2215612
```

```
t.test(resultsDW.fit$b.mfc.sr)
```

```
##
##  One Sample t-test
##
## data:  resultsDW.fit$b.mfc.sr
## t = 3.8293, df = 39, p-value = 0.0004546
```

```
## alternative hypothesis: true mean is not equal to 0
## 95 percent confidence interval:
##  0.1045289 0.3385935
## sample estimates:
## mean of x
## 0.2215612
```

```
(se.b <- sd(resultsDW.fit$b.mfc.sr)/sqrt(length(resultsDW.fit$b.mfc.sr)))
```

```
## [1] 0.0578597
```

```
hist(resultsDW.fit$b.mfc.sr, xlim=c(-1.7,1.4), xlab="slope sex ratio",
     main="corrected MF agr: slope sex ratio (DW)")
emp.res <- coef(summary(glmmTMBc1))$cond[2,1]
abline(v=emp.res, col="red",lwd=2)
text(x=emp.res+0.2, y=8, labels="empirical result", col="red")
```

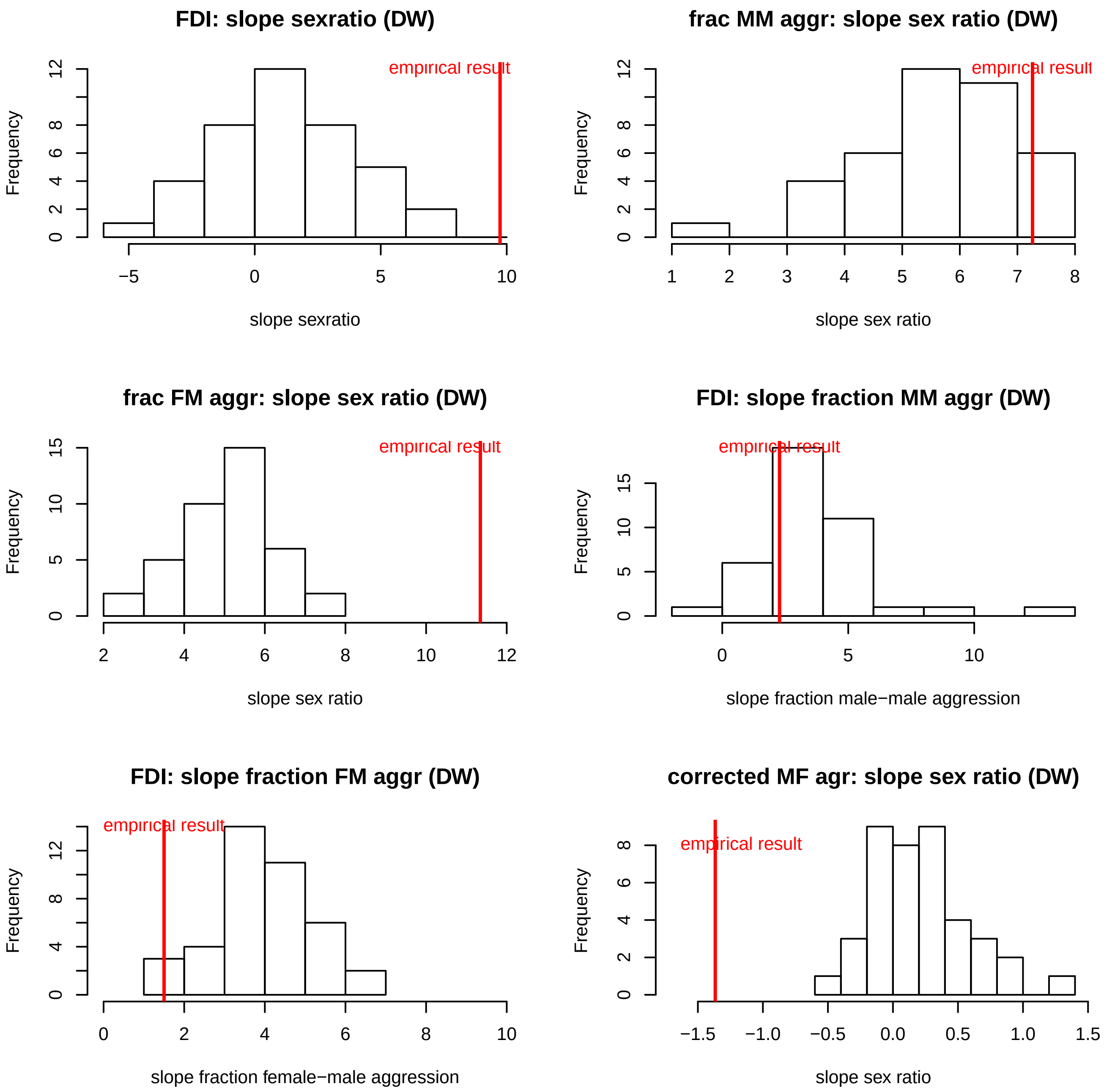

Empirical data (model 1): betabinomial GLMM for FDI explained by proportion of males

We analyze the Female Dominance Index using a Generalized Linear Mixed Model (GLMM), assuming a betabinomial distribution and logit link function.

The random part of the model consists of a random population effect (three populations: fbv, pecb, una) and a random group-within-population effect: within population fbv there are two groups (followed over time), within population pecb there are three groups (in single years) and within population una there is one group (in three years). The random effects of groups within populations handle repeated measurements over time (years). In all GLMMs we use the same random part of the model.

In the next analysis, the proportion of males (sexratio) enters as regressor the fixed part of the model.

```
glmmTMBo1 <- glmmTMB(cbind(FDI.num, FDI.denom-FDI.num) ~ sexratio + (1 | pop / group),
                     family=betabinomial,data=C)
logLik(glmmTMBo1)
```

```
## 'log Lik.' -28.39251 (df=5)
```

```
summary(glmmTMBo1)
```

```
##  Family: betabinomial  ( logit )
## Formula:
## cbind(FDI.num, FDI.denom - FDI.num) ~ sexratio + (1 | pop/group)
## Data: C
##
##      AIC      BIC   logLik deviance df.resid
##     66.8     70.0    -28.4     56.8        9
##
## Random effects:
##
## Conditional model:
##  Groups    Name        Variance  Std.Dev.
##  group:pop (Intercept) 2.460e-02 1.569e-01
##  pop       (Intercept) 3.076e-09 5.547e-05
## Number of obs: 14, groups:  group:pop, 6; pop, 3
##
## Overdispersion parameter for betabinomial family (): 33.2
##
## Conditional model:
##             Estimate Std. Error z value Pr(>|z|)
## (Intercept)   -4.446      1.012  -4.393 1.12e-05
## sexratio       9.736      2.341   4.158 3.21e-05
```

```
Anova(glmmTMBo1)
```

```
## Analysis of Deviance Table (Type II Wald chisquare tests)
##
## Response: cbind(FDI.num, FDI.denom - FDI.num)
##           Chisq Df Pr(>Chisq)
## sexratio 17.291  1  3.208e-05
```

```
# Some model diagnostics using DHARMa package
simulationOutput <- simulateResiduals(fittedModel = glmmTMBo1, n=10000)
plot(simulationOutput)
```

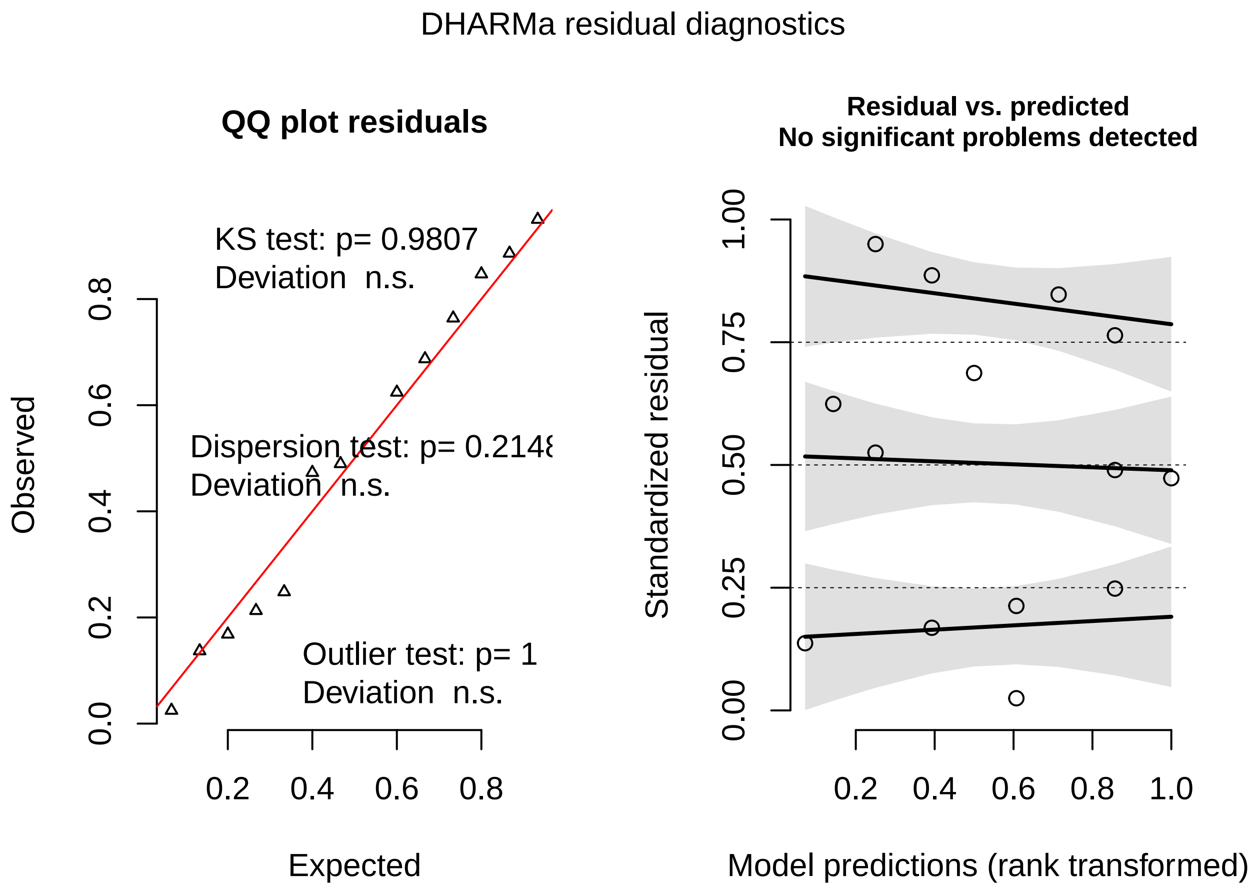


```
# Binomial overdispersion?
getRho <- function(obj){
  mm <- model.matrix(obj$modelInfo$allForm$dispformula,
                     data=obj$frame)
  fixdisp <- fixef(obj)[["disp"]]
  1/(1+exp(mm%*%fixdisp))
}
getRho(glmmTMBo1)[1]
```

```
## [1] 0.02928066
```

```
# rho is dispersion parameter:
# betabinomial distribution has variance var(k/n)=(P(1-P)/n)*(1+(n-1) rho)
# rho = 0 means binomial variance;
# here rho = 0.03, i.e. hardly binomial overdispersion.

# Plotting fitted model
cf <- fixef(glmmTMBo1)
V <- vcov(glmmTMBo1)
V <- V$cond

pred.at  <- data.frame(sexratio=seq(0.1,0.6, by=0.001), pop="fbv", group="CH")
eta <- predict(glmmTMBo1, pred.at)
phat <- 1/(1+exp(-eta))

L <- cbind(1, pred.at$sexratio)

var.eta <- diag(L %*% V %*% t(L))
se.eta <- sqrt(var.eta)
lwr <- 1/(1+exp(-(eta-1.96*se.eta)))
```

```r
upr <- 1/(1+exp(-(eta+1.96*se.eta)))

df <- data.frame(sexratio=pred.at$sexratio, phat=phat, lwr=lwr, upr=upr)

man.col <- c("#F8766D", "#7CAE00", "#C77CFF", "#00BFC4")

# Plot 95% confidence bands and points instead of lines
p <- ggplot(data=C, aes(x=sexratio, y=FDI)) +
  labs(x = "proportion of males", y = "female dominance index") +
  theme_bw()+ theme(panel.grid.minor = element_blank()) +
  geom_point(aes(colour=pop, group=pop), size=2) +
  guides(colour = guide_legend(override.aes = list(alpha = 1), title="population")) +
  scale_colour_manual(values=man.col) +

  geom_ribbon(data=df, mapping=aes(x=sexratio, y=phat, ymin = lwr, ymax = upr), fill="darkgrey", alpha = .2) +
  geom_line(data=df, mapping=aes(x=sexratio, y=phat),  size=0.8, colour="darkgrey")
p
```

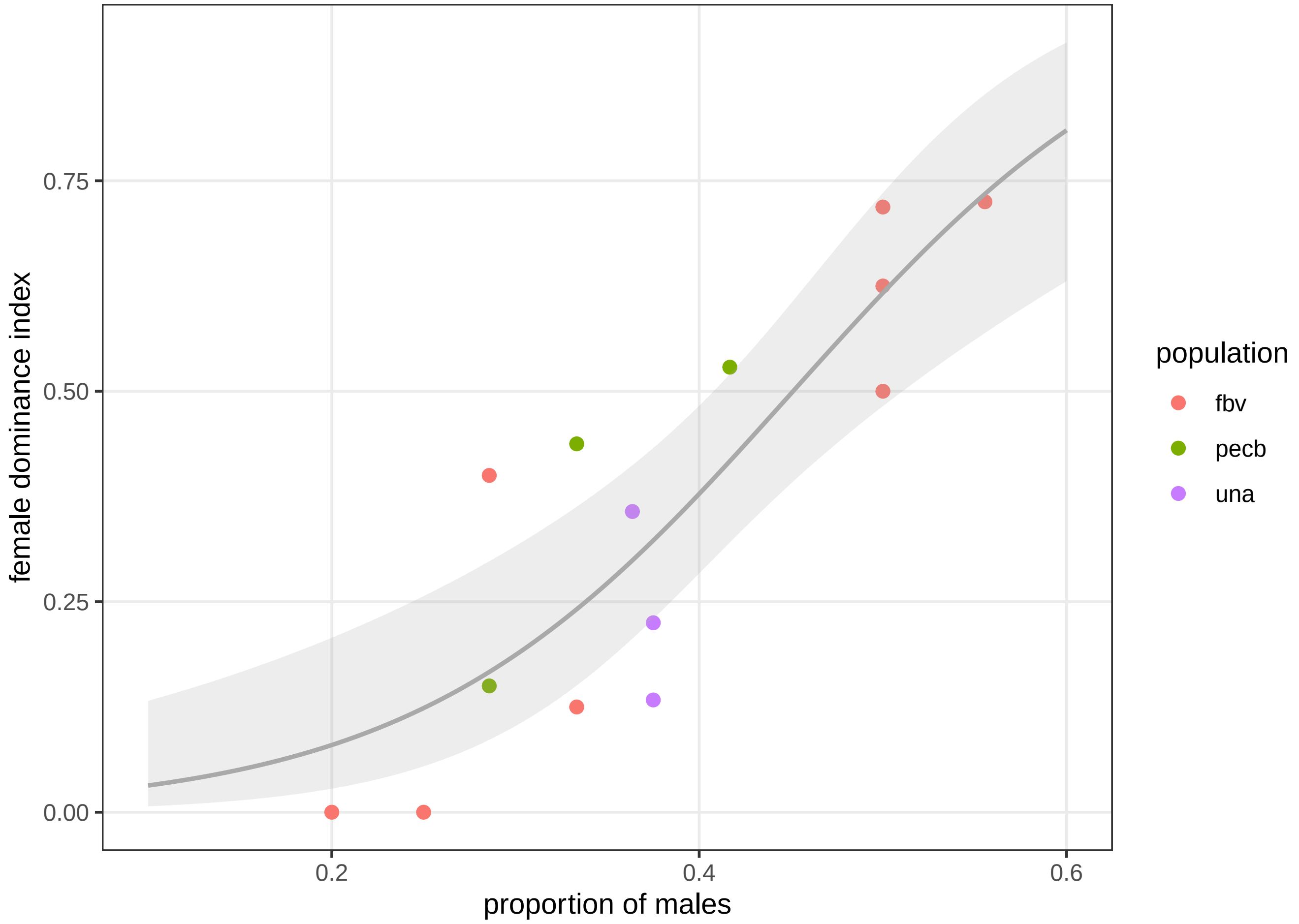


```r
#Null model of no effect at all
glmmTMBo0 <- glmmTMB(cbind(FDI.num, FDI.denom-FDI.num) ~ 1, family=betabinomial, data=C)
summary(glmmTMBo0)
```

```
##  Family: betabinomial  ( logit )
## Formula:          cbind(FDI.num, FDI.denom - FDI.num) ~ 1
## Data: C
##
##      AIC      BIC   logLik deviance df.resid
##     75.5     76.8    -35.8     71.5       12
##
```

```
##
## Overdispersion parameter for betabinomial family (): 6.01
##
## Conditional model:
##             Estimate Std. Error z value Pr(>|z|)
## (Intercept)  -0.4948     0.2509  -1.972   0.0486
```

```
logLik(glmmTMBo0)
```

```
## 'log Lik.' -35.76629 (df=2)
```

```
# Omnibus test:
anova(glmmTMBo1, glmmTMBo0, test="LRT")
```

```
## Data: C
## Models:
## glmmTMBo0: cbind(FDI.num, FDI.denom - FDI.num) ~ 1, zi=~0, disp=~1
## glmmTMBo1: cbind(FDI.num, FDI.denom - FDI.num) ~ sexratio + (1 | pop/group), zi=~0, disp=~1
##           Df    AIC    BIC  logLik deviance  Chisq Chi Df Pr(>Chisq)
## glmmTMBo0  2 75.533 76.811 -35.766   71.533
## glmmTMBo1  5 66.785 69.980 -28.393   56.785 14.748      3   0.002046
```

```
# There is some effect of sexratio and/or pop / group.

r.squaredLR(glmmTMBo1, null = glmmTMBo0) # Likelihood based R-square for sexratio and pop / group together
```

```
## [1] 0.6512489
## attr(,"adj.r.squared")
## [1] 0.6552058
```

```
# Effect of sexratio?
glmmTMBo2 <- glmmTMB(cbind(FDI.num, FDI.denom-FDI.num)  ~ 1 + (1 | pop / group), family=betabinomial, data=C)
anova(glmmTMBo1, glmmTMBo2, test="LRT")
```

```
## Data: C
## Models:
## glmmTMBo2: cbind(FDI.num, FDI.denom - FDI.num) ~ 1 + (1 | pop/group), zi=~0, disp=~1
## glmmTMBo1: cbind(FDI.num, FDI.denom - FDI.num) ~ sexratio + (1 | pop/group), zi=~0, disp=~1
##           Df    AIC    BIC  logLik deviance  Chisq Chi Df Pr(>Chisq)
## glmmTMBo2  4 79.533 82.089 -35.766   71.533
## glmmTMBo1  5 66.785 69.980 -28.393   56.785 14.748      1  0.0001229
```

```
# Likelihood ratio test for sexratio: P=0.0001229; strong relation of sexratio with FDI

r.squaredLR(glmmTMBo1, null = glmmTMBo2) # Likelihood based R-square for sexratio alone
```

```
## [1] 0.6512489
## attr(,"adj.r.squared")
## [1] 0.6552058
```

```
# Group does not explain anything extra.

# Random effect of pop / group?
glmmTMBo3 <- glmmTMB(cbind(FDI.num, FDI.denom-FDI.num)  ~ sexratio, family=betabinomial, data=C)
anova(glmmTMBo1, glmmTMBo3, test="LRT")
```

```
## Data: C
## Models:
## glmmTMBo3: cbind(FDI.num, FDI.denom - FDI.num) ~ sexratio, zi=~0, disp=~1
## glmmTMBo1: cbind(FDI.num, FDI.denom - FDI.num) ~ sexratio + (1 | pop/group), zi=~0, disp=~1
##           Df    AIC    BIC  logLik deviance  Chisq Chi Df Pr(>Chisq)
## glmmTMBo3  3 62.816 64.734 -28.408   56.816
## glmmTMBo1  5 66.785 69.980 -28.392   56.785 0.0314      2     0.9844
```

```
# Random effects of population and group within population unimportant.
(Pval.randomeffects <- 1 - (0.25*1 + 0.5*pchisq(0.0314,1) + 0.25*pchisq(0.0314,2)))  # P=0.68
```

```
## [1] 0.6757811
```

```
# Betabinomial overdispersion?
glmmTMBo4 <- glmmTMB(cbind(FDI.num, FDI.denom-FDI.num)  ~ sexratio + (1 | pop / group), family=binomial, data=
anova(glmmTMBo1, glmmTMBo4, test="LRT")
```

```
## Data: C
## Models:
## glmmTMBo4: cbind(FDI.num, FDI.denom - FDI.num) ~ sexratio + (1 | pop/group), zi=~0, disp=~1
## glmmTMBo1: cbind(FDI.num, FDI.denom - FDI.num) ~ sexratio + (1 | pop/group), zi=~0, disp=~1
##           Df    AIC    BIC  logLik deviance  Chisq Chi Df Pr(>Chisq)
## glmmTMBo4  4 66.291 68.848 -29.146   58.291
## glmmTMBo1  5 66.785 69.980 -28.392   56.785 1.5063      1     0.2197
```

```
# Binomial overdispersion is unimportant.
(Pval.overdisp <- 0.5*pchisq(1.5063,1, lower.tail=FALSE))  # P=0.11
```

```
## [1] 0.1098523
```

Empirical data (models 2, 3): betabinomial GLMM's for proportion MM aggression and proportion FM aggression explained by proportion of males

In the next analyses we check whether the proportion male-male aggression of total male aggression can be explained by proportion males (model 2), as well as whether the proportion female-male aggression of total female aggression can be explained by proportion males (model 3).

Again, we use betabinomial GLMM's, now for the proportion of male-male ago out of total male ago, regressing on proportion males, and for the proportion female-male ago out of total female ago, regressing on proportion males.

We keep the random part of the model as before.

```
# Explain fraction MM aggression (of total M aggression) by sexratio, N=12 (only groups with nmales>1)
glmmTMBe1 <- glmmTMB(cbind(MMago, MFago) ~ sexratio + (1 | pop / group),
                       family=betabinomial, data=C[C$nmales>1,])
summary(glmmTMBe1)
```

```
##  Family: betabinomial  ( logit )
## Formula:          cbind(MMago, MFago) ~ sexratio + (1 | pop/group)
## Data: C[C$nmales > 1, ]
##
##      AIC      BIC   logLik deviance df.resid
##     79.8     82.2    -34.9     69.8        7
##
## Random effects:
##
## Conditional model:
##  Groups    Name        Variance  Std.Dev.
##  group:pop (Intercept) 8.278e-10 2.877e-05
##  pop       (Intercept) 5.317e-01 7.292e-01
## Number of obs: 12, groups:  group:pop, 6; pop, 3
##
## Overdispersion parameter for betabinomial family (): 17.2
##
## Conditional model:
##             Estimate Std. Error z value Pr(>|z|)
## (Intercept)   -2.677      1.172  -2.284  0.02235
## sexratio       7.262      2.784   2.609  0.00909
```

```
logLik(glmmTMBe1)
```

```
## 'log Lik.' -34.90514 (df=5)
```

```
# Explain fraction FM aggression (of total F aggression) from sexratio
glmmTMBe2 <- glmmTMB(cbind(FMago, FFago) ~ sexratio + (1 | pop / group),
                       family=betabinomial, data=C)
summary(glmmTMBe2)
```

```
##  Family: betabinomial  ( logit )
## Formula:          cbind(FMago, FFago) ~ sexratio + (1 | pop/group)
## Data: C
##
##      AIC      BIC   logLik deviance df.resid
##     75.4     78.5    -32.7     65.4        9
##
## Random effects:
##
## Conditional model:
##  Groups    Name        Variance  Std.Dev.
##  group:pop (Intercept) 1.478e-05 0.003844
##  pop       (Intercept) 1.160e-06 0.001077
## Number of obs: 14, groups:  group:pop, 6; pop, 3
##
```

```
## Overdispersion parameter for betabinomial family (): 5.58
##
## Conditional model:
##             Estimate Std. Error z value Pr(>|z|)
## (Intercept)   -4.804      1.211  -3.966  7.3e-05
## sexratio      11.346      3.040   3.732  0.00019
```

```r
logLik(glmmTMBe2)
```

```
## 'log Lik.' -32.6752 (df=5)
```

Empirical data (models 4, 5): betabinomial GLMM for FDI explained by proportion MM aggression and proportion FM aggression

In the next analysis we keep the Female Dominance Index as response in the GLMM and we keep the same random part of the model, but we check whether the FDI can be explained by other fixed-effect regressors. We investigate the following relationships:

model 4: FDI vs proportion of male-male aggression of all male aggression

model 5: FDI vs proportion of female-male aggression of all female aggression

```
# Can FDI be explained from fraction male-male aggression and fraction female-male aggression?

C$MMagopertotMago <- C$MMago/(C$MMago+C$MFago)
C$FMagopertotFago <- C$FMago/(C$FFago+C$FMago)

# Explain FDI from MMagopertotMago, N=12 (only groups with nmales>1)
# Model with MM aggr per total M aggr alone
glmmTMBa1 <- glmmTMB(cbind(FDI.num, FDI.denom-FDI.num) ~ MMagopertotMago + (1 | pop / group),
                     family=betabinomial, data=C[C$nmales>1,])
summary(glmmTMBa1)
```

```
##  Family: betabinomial  ( logit )
## Formula:
## cbind(FDI.num, FDI.denom - FDI.num) ~ MMagopertotMago + (1 |
##     pop/group)
## Data: C[C$nmales > 1, ]
##
##      AIC      BIC   logLik deviance df.resid
##     70.8     73.3    -30.4     60.8        7
##
## Random effects:
##
## Conditional model:
##  Groups    Name        Variance  Std.Dev.
##  group:pop (Intercept) 9.391e-10 3.064e-05
##  pop       (Intercept) 2.814e-01 5.305e-01
## Number of obs: 12, groups:  group:pop, 6; pop, 3
##
## Overdispersion parameter for betabinomial family (): 40.8
##
## Conditional model:
##                 Estimate Std. Error z value Pr(>|z|)
## (Intercept)      -1.7005     0.6504  -2.615  0.00893
## MMagopertotMago   2.2729     0.9832   2.312  0.02079
```

```
logLik(glmmTMBa1)
```

```
## 'log Lik.' -30.41866 (df=5)
```

```
# Explain FDI from FMagopertotFago, N=14
# Model with FM aggr per total female aggr
glmmTMBa2 <- glmmTMB(cbind(FDI.num, FDI.denom-FDI.num) ~ FMagopertotFago + (1 | pop / group),
                     family=betabinomial, data=C)
summary(glmmTMBa2)
```

```
##  Family: betabinomial  ( logit )
## Formula:
## cbind(FDI.num, FDI.denom - FDI.num) ~ FMagopertotFago + (1 |
##     pop/group)
## Data: C
##
##      AIC      BIC   logLik deviance df.resid
```

```
##     79.0     82.2    -34.5     69.0        9
##
## Random effects:
##
## Conditional model:
##  Groups    Name        Variance  Std.Dev.
##  group:pop (Intercept) 1.961e-08 0.00014
##  pop       (Intercept) 3.160e-02 0.17777
## Number of obs: 14, groups:  group:pop, 6; pop, 3
##
## Overdispersion parameter for betabinomial family (): 6.77
##
## Conditional model:
##                 Estimate Std. Error z value Pr(>|z|)
## (Intercept)      -1.1834     0.5611  -2.109   0.0349
## FMagopertotFago   1.5012     1.0007   1.500   0.1336
```

```
logLik(glmmTMBa2)
```

```
## 'log Lik.' -34.49675 (df=5)
```

Empirical data (model 6): negative binomial GLMM for MF aggression, corrected for gender chance aggression, explained by proportion of males

In this analysis for the empirical data we check whether there are signs supporting the Docile Male Hypothesis. For this, we relate the frequency of male-female aggression to the proportion males, but now correcting for the total frequency of male agonistic activities and the proportion of female opponents of the aggressor (see earlier explanation under analysis of DomWorld data).

Below we select only those groups with at least two males ($n = 12$).

```
C$offsmf <- log((C$MMago+C$MFago)*(C$nfemales/(C$nadults-1)))

glmmTMBc1 <- glmmTMB(MFago ~ offset(offsmf) + sexratio + (1 | pop / group),
                     family=nbinom2(link="log"), data=C[C$nmales>1,])
summary(glmmTMBc1)
```

```
##  Family: nbinom2  ( log )
## Formula:          MFago ~ offset(offsmf) + sexratio + (1 | pop/group)
## Data: C[C$nmales > 1, ]
##
##      AIC      BIC   logLik deviance df.resid
##     83.5     85.9    -36.7     73.5        7
##
## Random effects:
##
## Conditional model:
##  Groups    Name        Variance  Std.Dev.
##  group:pop (Intercept) 2.605e-10 1.614e-05
##  pop       (Intercept) 1.283e-01 3.581e-01
## Number of obs: 12, groups:  group:pop, 6; pop, 3
##
## Overdispersion parameter for nbinom2 family (): 20.1
##
## Conditional model:
##             Estimate Std. Error z value Pr(>|z|)
## (Intercept)   0.1086     0.5857   0.185    0.853
## sexratio     -1.3664     1.4174  -0.964    0.335
```

```
logLik(glmmTMBc1)
```

```
## 'log Lik.' -36.73494 (df=5)
```

We find a negative regression coefficient, but with $P = 0.34$. Therefore, there is no evidence found for the Docile Male Hypothesis here.

Empirical data (model 7): betabinomial GLMM for FDI explained by absolute number of males

In this final analysis of the empirical data, we analyze again the FDI with a betabinomial distribution, with the same random part of the model as before, but with the absolute number of males as regressor instead of the proportion of males.

How do the AIC's of model 1 and model 7 compare?

```
# Is Female Dominance Index better explained by absolute number of males compared to proportion of males?
glmmTMBo5 <- glmmTMB(cbind(FDI.num, FDI.denom-FDI.num) ~ nmales + (1 | pop / group),
                     family=betabinomial,data=C)
AIC(glmmTMBo5, glmmTMBo1)
```

```
##           df      AIC
## glmmTMBo5  5 78.83695
## glmmTMBo1  5 66.78503
```

The AIC of model with nmales as regressor is 12.1 higher than AIC of model with sexratio as regressor. We conclude that the model with absolute number of males as regressor fits worse.
